# Xiexin Tang improves the symptom of type 2 diabetic rats by modulation of the gut microbiota

**DOI:** 10.1038/s41598-018-22094-2

**Published:** 2018-02-27

**Authors:** Xiaoyan Wei, Jinhua Tao, Suwei Xiao, Shu Jiang, Erxin Shang, Zhenhua Zhu, Dawei Qian, Jinao Duan

**Affiliations:** Jiangsu Collaborative Innovation Center of Chinese Medicinal Resources Industrialization, Nanjing University of Chinese Medicine, 138 Xianlin Road, Nanjing, 210023 PR China

## Abstract

Type 2 diabetes mellitus (T2DM), a chronic metabolic disease which severely impairs peoples’ quality of life, currently attracted worldwide concerns. There are growing evidences that gut microbiota can exert a great impact on the development of T2DM. Xiexin Tang (XXT), a traditional Chinese medicine prescription, has been clinically used to treat diabetes for thousands of years. However, few researches are investigated on the modulation of gut microbiota community by XXT which will be very helpful to unravel how it works. In this study, bacterial communities were analyzed based on high-throughput 16S rRNA gene sequencing. Results indicated that XXT could notably shape the gut microbiota. T2DM rats treated with XXT exhibited obvious changes in the composition of the gut microbiota, especially for some short chain fatty acids producing and anti-inflammatory bacteria such as *Adlercreutzia*, *Alloprevotella*, *Barnesiella*, [*Eubacterium*] *Ventriosum group*, *Blautia*, *Lachnospiraceae UCG-001*, *Papillibacter* and *Prevotellaceae NK3B31 group*. Additionally, XXT could also significantly ameliorate hyperglycemia, lipid metabolism dysfunction and inflammation in T2DM rats. Moreover, the correlation analysis illustrated that the key microbiota had a close relationship with the T2DM related indexes. The results probably provided useful information for further investigation on its active mechanism and clinical application.

## Introduction

T2DM, a chronic metabolic disease characterized by hyperglycemia as a result of insufficient insulin secretion, insulin action or both^1^, is estimated that its numbers in the adults will increase by 55% by 2035^2^. Its currently high global prevalence will result in a growing burden^3^. Therefore, prevention and treatment of T2DM have attracted great attentions in the past decades and become a matter of global research interest. In the past years, many effective hypoglycemic agents like α-glucosidase inhibitor, insulin secretagogue, insulin sensitizer and intestinal lipase inhibitor have been developed for the treatment of T2DM^4–6^. However, these drugs, which can effectively reduce the level of blood glucose, have a series of adverse effects and complications such as hypoglycemia, gastrointestinal discomfort and pulmonary edema, etc^7,8^. Thus, traditional Chinese herb medicines characterized with a systematic and integral regulation by multi-active components, multi-paths and multi-targets are becoming the significant sources for the expansion of anti-diabetic drugs^9–11^.

With thousands of years of medical practice, a large number of valuable experiences have been accumulated in the traditional Chinese medical system for diabetes therapy and many are still in present use^10^. Some of classical prescriptions (such as *Tangmaikang Jiaonang*, *Xiaoke Wan*, *Xiaotangling Jiaonang* and *Yuquan Wan*, etc^10^.) have been successfully developed into preparing for treatment of diabetes according to the progress of medicinal technology, which performed well. Xiexin Tang (XXT) originating from the Medical Treasures of the Golden Chamber written by Zhang Zhongjing is a compound recipe with heat-clearing and detoxication. This classic prescription composed of Dahuang (*Rhei rhizome*), Huangqin (*Scutellaria radix*) and Huanglian (*Coptidis rhizome*), which has some potentially beneficial effects such as anti-inflammation^12^, anti-apoptosis^13^, neuron protection^14^, anti-oxidation^15^ and immunomodulation^16^, is clinically used to treat constipation, high fever, restlessness and insomnia. Furthermore, it has been frequently applied to cure diabetic mellitus (called *Xiaokezheng* or *Xiaodanzheng* in the traditional Chinese medical system) with remarkably therapeutic effects since the Tong Dynasty (6^th^ century C.E.)^17^. Numerous researches suggested that XXT possessed protective effects on the increase of blood glucose and dyslipidemia in high calorie feeding and STZ-induced diabetic rats, and was superior to metformin in improving insulin resistance index and reducing the lever of inflammatory factors^18,19^. The decoction showed the effect of anti-diabetic nephropathy by inhibiting the inflammation mediated by NF-κB^20^. Furthermore, many components of XXT could improve insulin resistance via several different pathways. For example, berberine ameliorated insulin resistance through suppressing the activation of M1 macrophage in adipose tissue^21^. Rhein could inhibit the yield of HFD-induced plasma LPS and the accumulation of pro-inflammatory macrophage^22^. Baicalin, one of the key components in *Scutellariae radix*, demonstrated antioxidant properties and hypoglycemic activity in diabetic rats^23^. However, the hypoglycemic mechanism of XXT by modulation of the gut microbiota is not clear up to now.

Recently, the major interest in the mechanism of T2DM and its therapeutic drugs has focused on the composition of human or animal gut microbiota^24–26^. It has been established that the gut microbiota, an essential part of the complicated host ecosystem, has associated with lots of human diseases like diabetes, obesity, inflammatory bowel diseases, liver cirrhosis, and etc^24,27–31^. Some papers reported that gut microbiota or their metabolites could affect energy balance^32^, glucose metabolism^33,34^, and low-grade inflammation^35^, which were closely associated with T2DM. A number of studies have exhibited that some components of XXT could attenuate insulin resistance by adjusting gut microbiota structure. Emodin could improve chronic kidney disease by reducing the number of harmful bacteria and altering the gut microbiota structure^36^. Oral administration of berberine could modify the mice gut microbiota composition to increase the producing of butyrate, which then entered blood and reduced glycolipid levels^37^. Thus, the gut microbiota might be a potential drug target for XXT to improve T2DM.

Whether the gut microbiota was a therapeutic target of XXT during improvement of T2DM symptoms, the effects of XXT on the gut microbial community in T2DM rats were investigated and key genera closely related to XXT treatment were identified by high-throughput 16S rRNA gene sequencing technology in this paper. Furthermore, the correlation analysis between gut flora and T2DM-related metabolic factors was carried out to explore the possible mechanism of XXT in improving T2DM.

## Results

### Effects of XXT on body weight and metabolic parameters of T2DM rats

**Figure 1:**
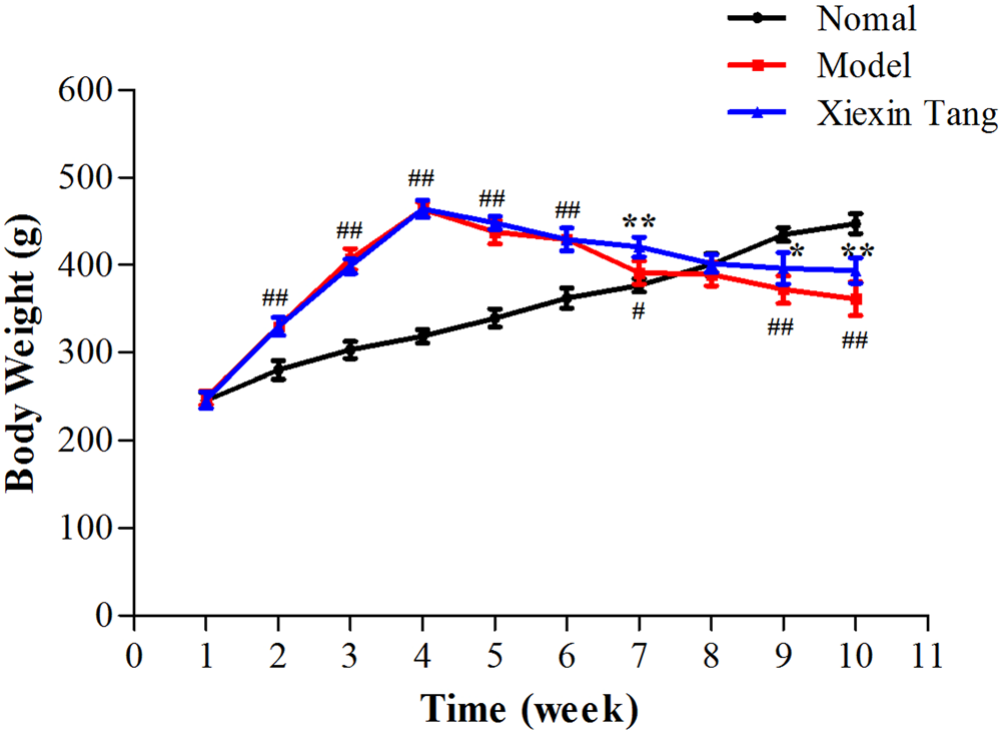
Effects of XXT on body weight of T2DM rats. Body weight over the 10 week experiments. The black line represented normal rats; the red line represented T2DM rats; the blue line represented XXT-treated T2DM rats. Results were expressed as mean ± SD, n = 6. ^##^P < 0.01, ^#^P < 0.05 vs. Control; ^**^P < 0.01, ^*^P < 0.05 vs. Model.

**Figure 2:**
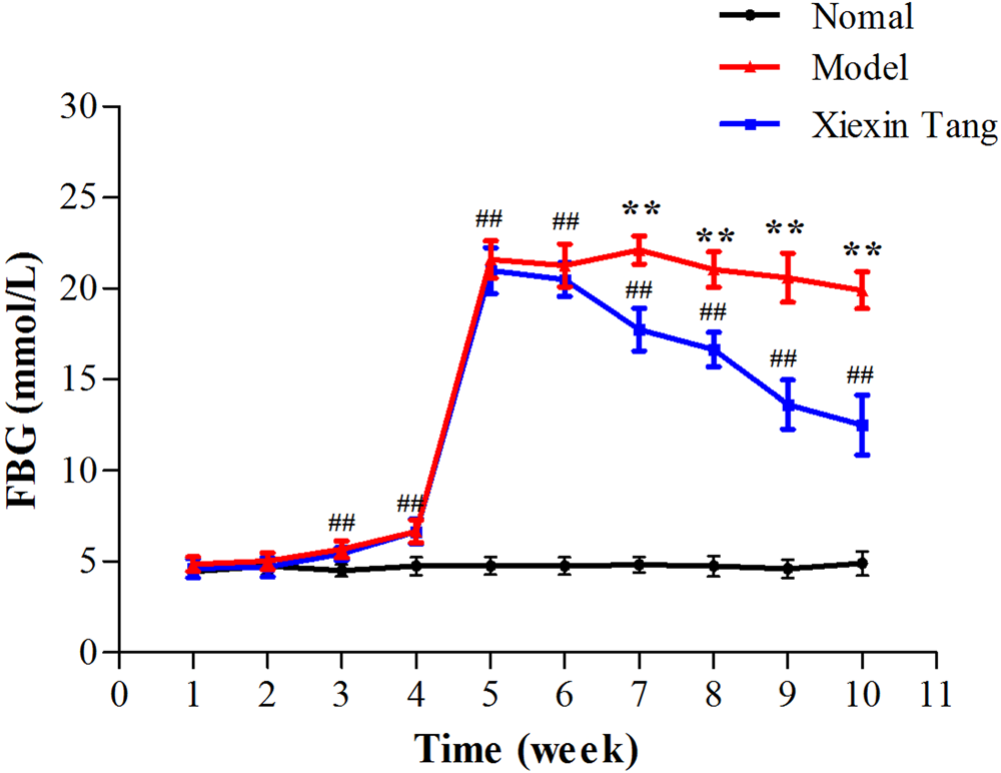
Effects of XXT on fasting blood glucose of T2DM rats. FBG over the 10 week experiments. The black line represented normal rats; the red line represented T2DM rats; the blue line represented XXT-treated T2DM rats. Results were expressed as mean ± SD, n = 6. ^##^P < 0.01, ^#^P < 0.05 vs. Control; ^**^P < 0.01, ^*^P < 0.05 vs. Model.

Body weights were standardized in all groups before experiment. As expected, the high fat diet (HFD) fed rats gained more weight than controls. After intraperitoneal injection of streptozotocin (STZ), the body weight of diabetic rats began to significantly reduced, while it kept increasing in the control group (Fig. 1). STZ-induced diabetic rats showed significant hyperglycemia compared with normal rats. Compared with the T2DM group, the FBG levels of rats in XXT-treated group were notably decreased. XXT exhibited a remarkable reduction of blood glucose and obvious impacts on body weight (Figs 1 and 2). Moreover, we also explored the shift of lipids and some inflammatory factors in T2DM rats, and the influences of XXT on these changes. The results indicated that T2DM rats had notably higher serum concentrations of low-density lipoprotein cholesterol (LDL-C), total cholesterol (TC) and triglycerides (TG), tumor necrosis factor-α (TNF-α), interleukin-6 (IL-6), C-reactive protein (CRP) and resistin compared to normal rats. While concentrations of high-density lipoprotein cholesterol (HDL-C) were highly lower in T2DM rats than in normal rats. However, the intake of XXT remarkably alleviated systematic inflammation and helped correct lipid metabolism disorders. The fasting insulin (FINS) levels in T2DM rats were obviously lower than that in the control group (*P* < 0.05), and not obviously different compared with XXT-treated group rats (Table 1). Furthermore, the levels of HOMR-IR and HOMR-ISI in three groups suggested that XXT could significantly improve insulin sensitivity and insulin resistance.

**Table 1:** Comparison of metabolic index levels in rats among the different groups. (mean ± SD, n = 6).

Group	Normal	Model	XXT
TNF-α (pg/mL)	195.54 ± 5.69	235.168 ± 11.35^##^	217.226 ± 1.99^*^
IL-6 (pg/mL)	110.01 ± 3.13	134.18 ± 9.56^##^	119.45 ± 1.01^**^
CRP (ng/mL)	1567.89 ± 59.89	1798.08 ± 49.91^##^	1602.86 ± 61.34^**^
Resistin (ng/mL)	56.03 ± 2.17	64.38 ± 4.19^##^	53.96 ± 3.63^**^
TG (mmol/L)	0.252 ± 0.005	0.400 ± 0.07^##^	0.274 ± 0.008^**^
TC (mmol/L)	0.709 ± 0.065	1.445 ± 0.186^##^	0.906 ± 0.163^**^
LDL-C (mmol/L)	1.023 ± 0.098	1.397 ± 0.128^##^	1.230 ± 0.087^*^
HDL-C (mmol/L)	0.9249 ± 0.200	0.3983 ± 0.086^##^	0.5400 ± 0.037^*^
FINS (mU/L)	35.41 ± 0.24	31.20 ± 1.07^#^	31.89 ± 0.52
HOMA-IR	7.712 ± 1.02	28.205 ± 1.44^##^	17.333 ± 2.29^**^
HOMA-ISI	−5.14 ± 0.13	−6.44 ± 0.05^##^	−5.95 ± 0.14^**^

^##^P < 0.01, ^#^P < 0.05 vs^.^ Control; ^**^P < 0.01, ^*^P < 0.05 vs. Model.

### Comparison of the gut microflora community composition among different groups and intervention of XXT

**Figure 3:**
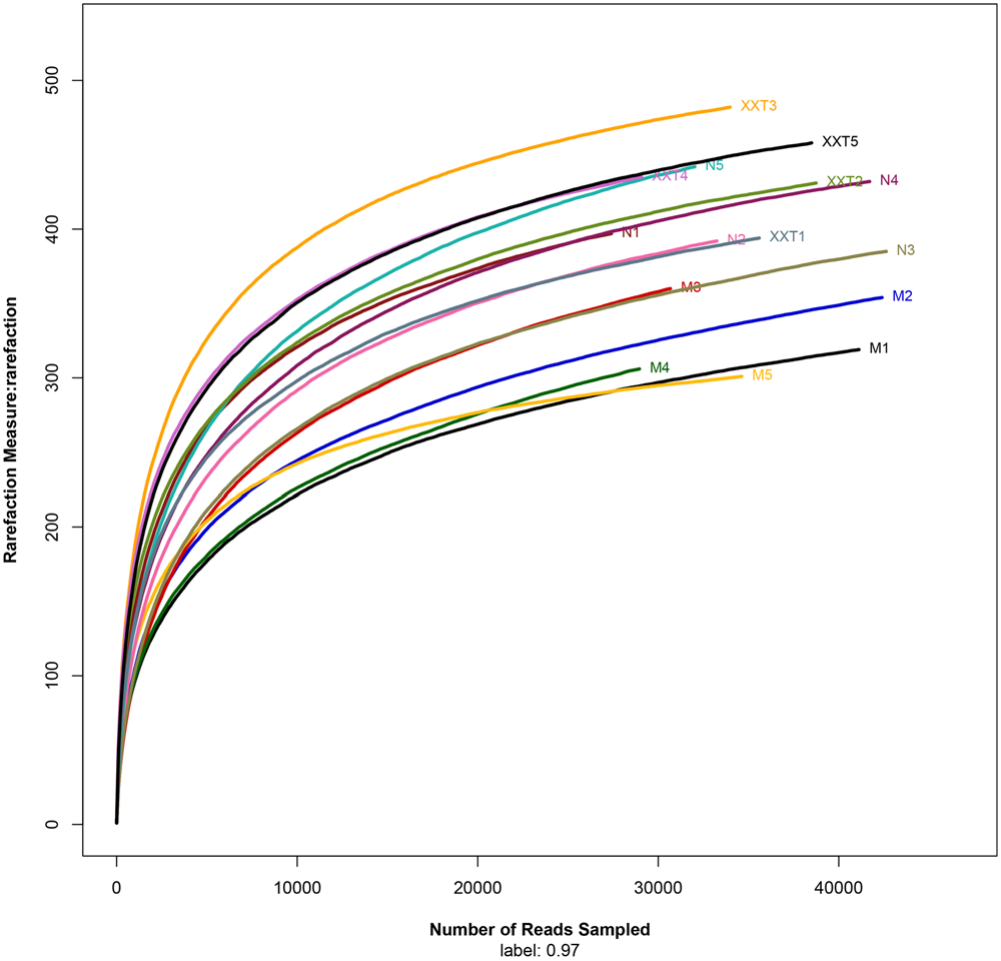
Rarefaction curves. Rarefaction analysis of V4-V5 pyrosequencing tags of the 16S rRNA gene in gut microbiota. N = normal; M = model; XXT = Xiexin Tang, n = 5.

**Figure 4:**
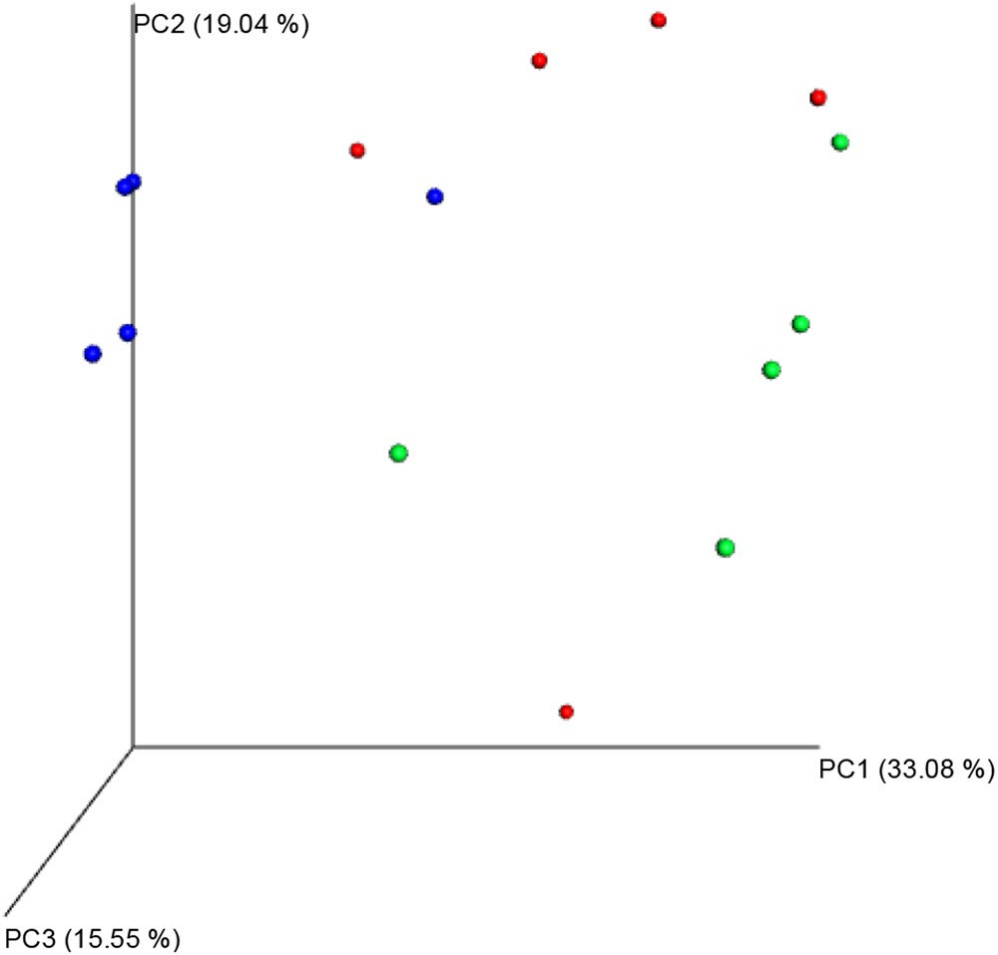
Principal coordination analysis of gut microbiota composition based on unweighted UniFrac in rats. Blue = normal; Red = model; Green = Xiexin Tang, n = 5.

A total of 530681 quality reads of 15 samples were generated with an average of 35378 ± 5215 reads per sample. The tendency of individual rarefaction curves indicated that the high sampling coverage was achieved in all samples (Fig. 3). As a brief summary and the estimators of diversity, it also illustrated that there were remarkable differences among normal, model and XXT-treated groups. Significant higher diversity was found in XXT-treated rats compared to normal or diabetic rat groups. The principal co-ordinates analysis (PCoA) of the unweighted Unifrac distances was used to compare the overall microbiota structures in three conditions. The result revealed an apparent separation in gut bacterial structure among three groups. The three principal component scores accounted for 33.08%, 19.04%, and 15.55% of total variations, respectively (Fig. 4).

**Figure 5:**
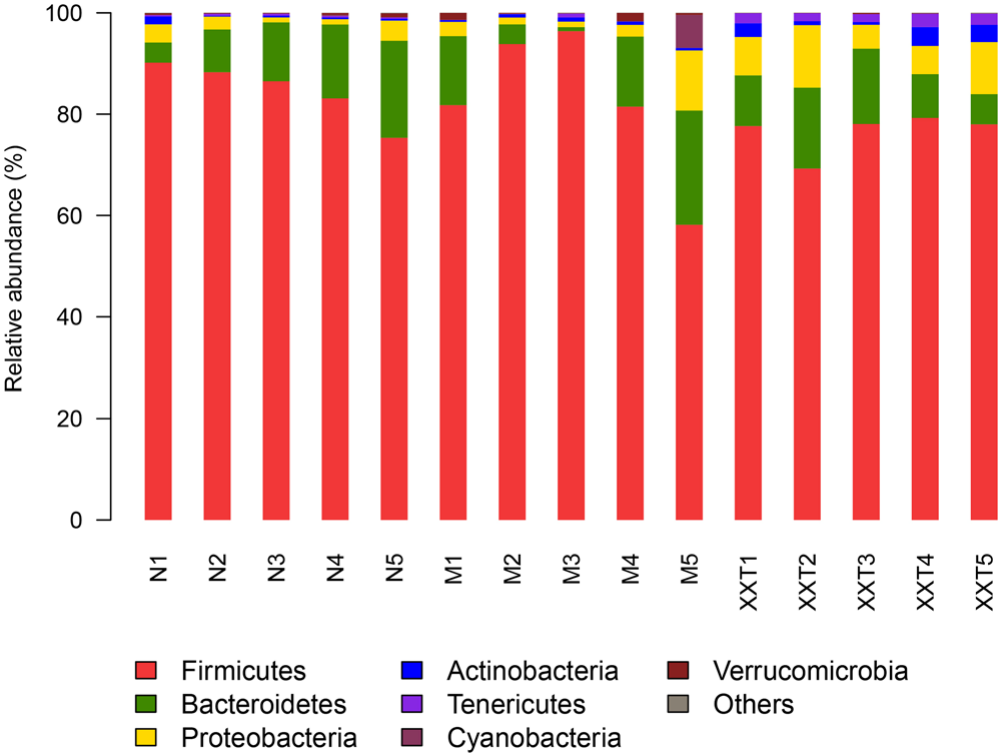
Relative abundance at the phylum level. N = normal; M = model, XXT = Xiexin Tang, n = 5.

Additionally, the microbial diversity analysis indicated that the gut microbiota included seven major phyla: *Firmicutes*, *Actinobacteria*, *Verrucomicrobia*, *Bacteroidetes*, *Tenericutes*, *Proteobacteria* and *Cyanobacteria* (Fig. 5). *Firmicutes* was the most abundant phyla in all samples. Samples from normal and model rats were both associated with a bloom of *Proteobacteria*, while a reduction of *Bacteroidetes* and an increase of *Firmicutes* in model rats were detected comparing with normal rats. However, XXT remarkably enriched the amount of *Proteobacteria* and *Actinobacteria*, and modulated the amount of *Bacteroidetes* and *Firmicutes*.

**Figure 6:**
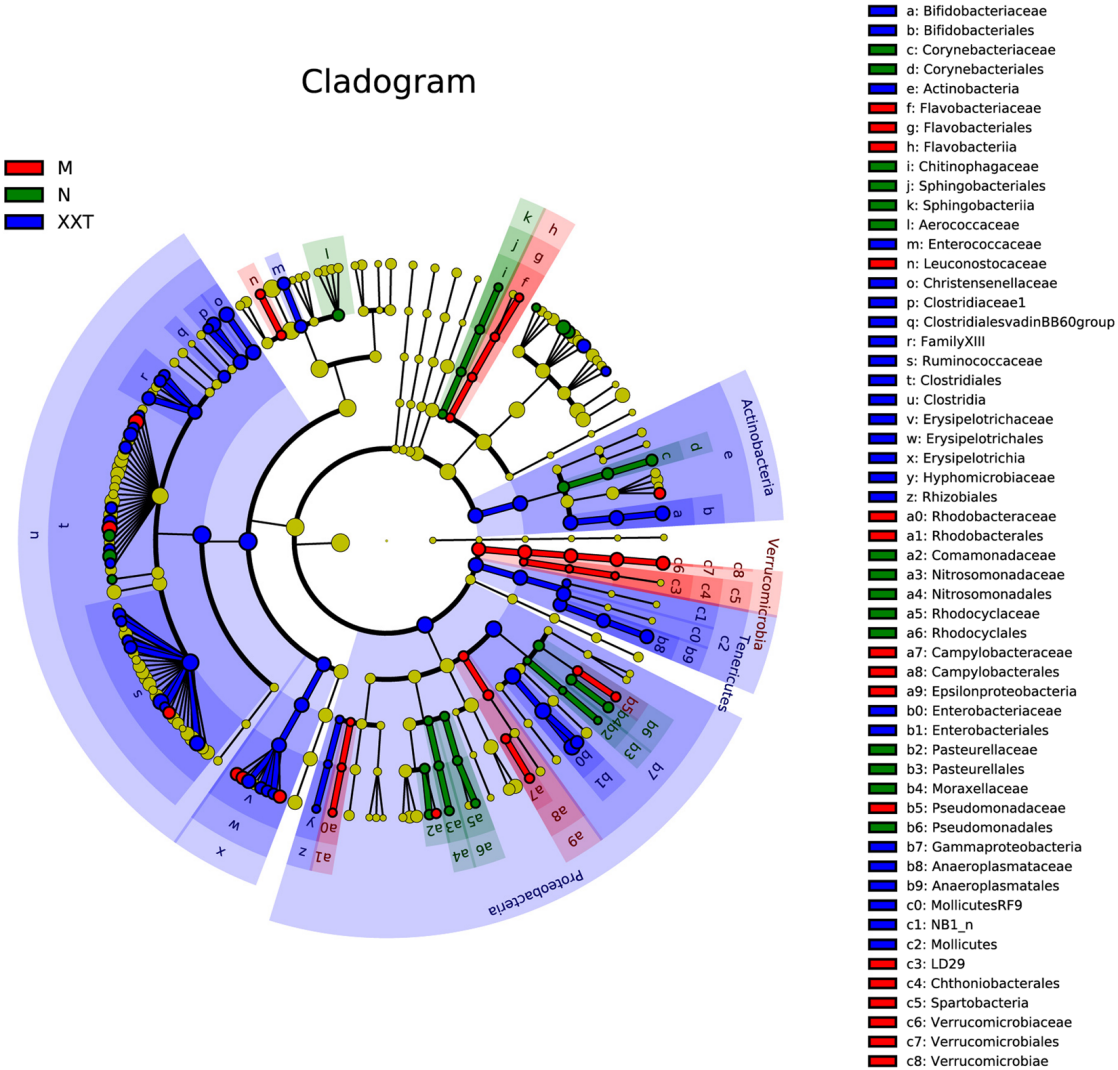
LEfSe comparison of gut microbiota among normal, model and Xiexin Tang-treated groups. Taxonomic cladogram derived from LEfSe analysis of 16S sequences. Blue = normal; Red = model; Green = Xiexin Tang, n = 5.

To identify the specific bacterial taxa in every condition, the composition of the microbiota from normal, T2DM and XXT-treated samples were compared by linear discriminant analysis effect size (LEfSe) method (Fig. 6 and Extended Data Figure S1). The constitutions of intestinal bacterial species among three groups showed significant variations. Twenty-eight key genera, among which several genera of *Proteobacteria* and *Coryebacteriaceae* of *Actinobacteria* were the most ones, were detected in the control group. While the most from 31 major genera recovered in the T2DM group belonged to *Proteobacteria* and *Verrucomicrobia*. Furthermore, fifty-nine main genera were restored in the XXT-treated group, and the most of these genera originated from *Proteobacteria*, *Actinobacteria*, *Verrucomicrobia* and *Clostridia*, respectively.

At genus level, 31 intestinal bacteria were exhibited significantly different between normal and T2DM rats in our study, and eight of them were markedly changed by XXT. The gut microbiota: *Alloprevotella*, *Barnesiella*, [*Eubacterium*] *Ventriosum group*, *Lachnospiraceae UCG-001*, *Papillibacter* and *Prevotellaceae NK3B31 group*, which obviously decreased in T2DM rat samples, were enriched in the XXT-treated group, while *Adlercreutzia* and *Blautia* were absolutely contrary in three groups (Table 2).

**Table 2:** Composition of microbiota genera in normal, model and XXT-treated groups after the 10 week experiments.

Classification levels of bacteria			Groups		
Phylum	Family	Genus	Normal	Model	XXT
Firmicutes	Acidaminococcaceae	*Papillibacter*	20 ± 3.5	2.6 ± 0.9^#^	105.6 ± 14.8^**^
	Eubacteriaceae	[*Eubacterium*] *Ventriosum group*	2.6 ± 1.1	0.4 ± 0.5^#^	9 ± 1.7^**^
	Lachnospiraceae	*Blautia*	19.8 ± 4.7	1540 ± 106.9^##^	793.2 ± 68.1^*^
		*Lachnospiraceae UCG-001*	42.2 ± 6.2	1.8 ± 0.2^##^	40 ± 11.3^**^
Bacteroidetes	Prevotellaceae	*Prevotellaceae NK3B31*	107 ± 12.8	16.4 ± 3.7^##^	86.6 ± 12.6^**^
		*Alloprevotella*	112.4 ± 10.5	22.8 ± 7.5^##^	244.2 ± 56.8^**^
	Porphyromonadaceae	*Barnesiella*	2.4 ± 0.6	0.6 ± 0.5^#^	5.8 ± 2.0^*^
Actinobacteria	Coriobacteriaceae	*Adlercreutzia*	3.4 ± 1.2	32 ± 9.8^#^	1.8 ± 0.3^**^

^##^P < 0.01, ^#^P < 0.05 vs^.^ Control; ^**^P < 0.01, ^*^P < 0.05 vs. Model.

### Correlation of gut microflora with metabolic parameters of T2DM

**Figure 7:**
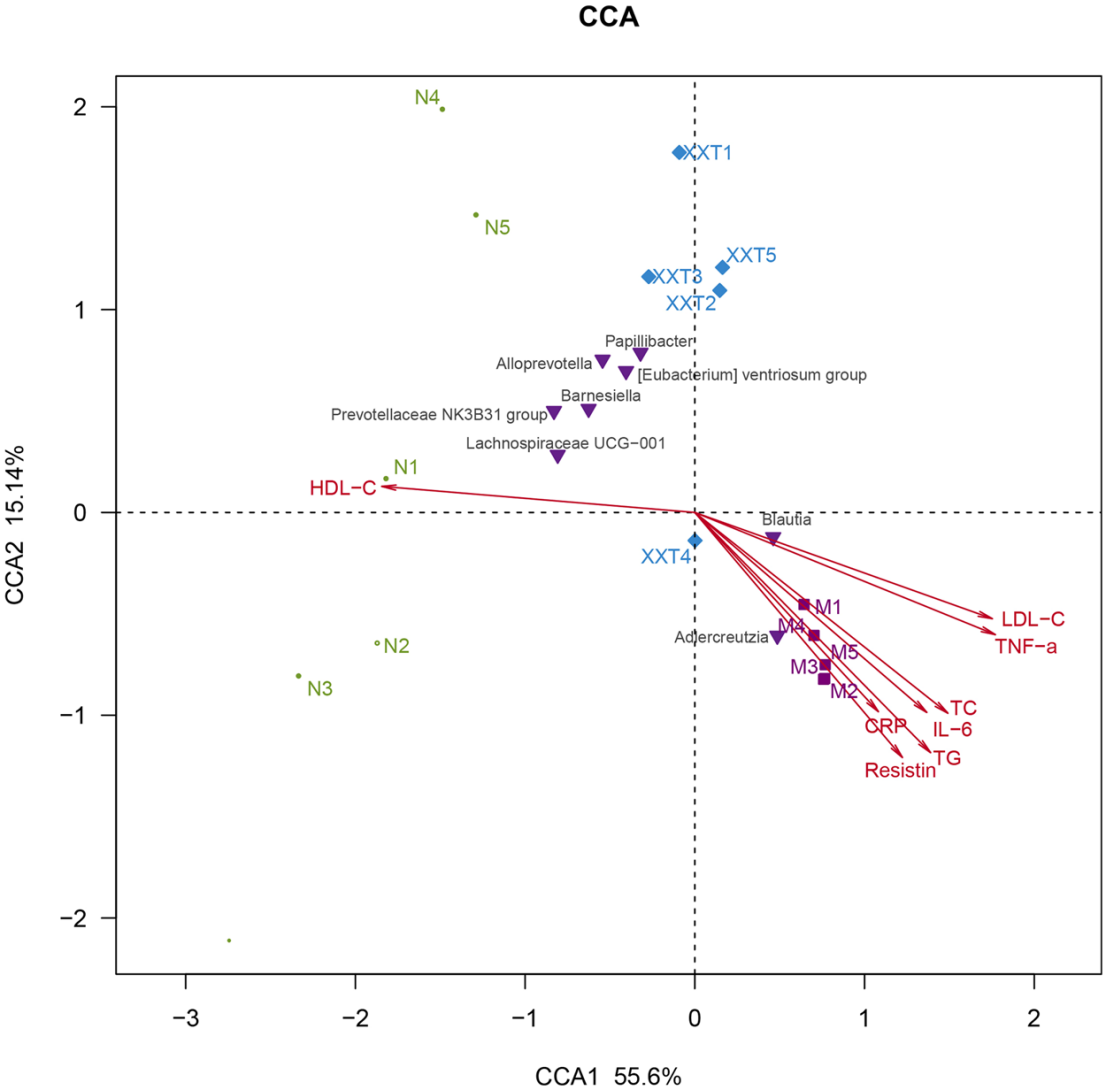
Correlation analysis between T2DM-related biochemical factors and gut microflora. Canonical correspondence analysis (CCA). The colors ranged from orange (negative correlation) to green (positive correlation). Significant correlations were noted by ^*^P < 0.05 and ^**^P < 0.01.

**Figure 8:**
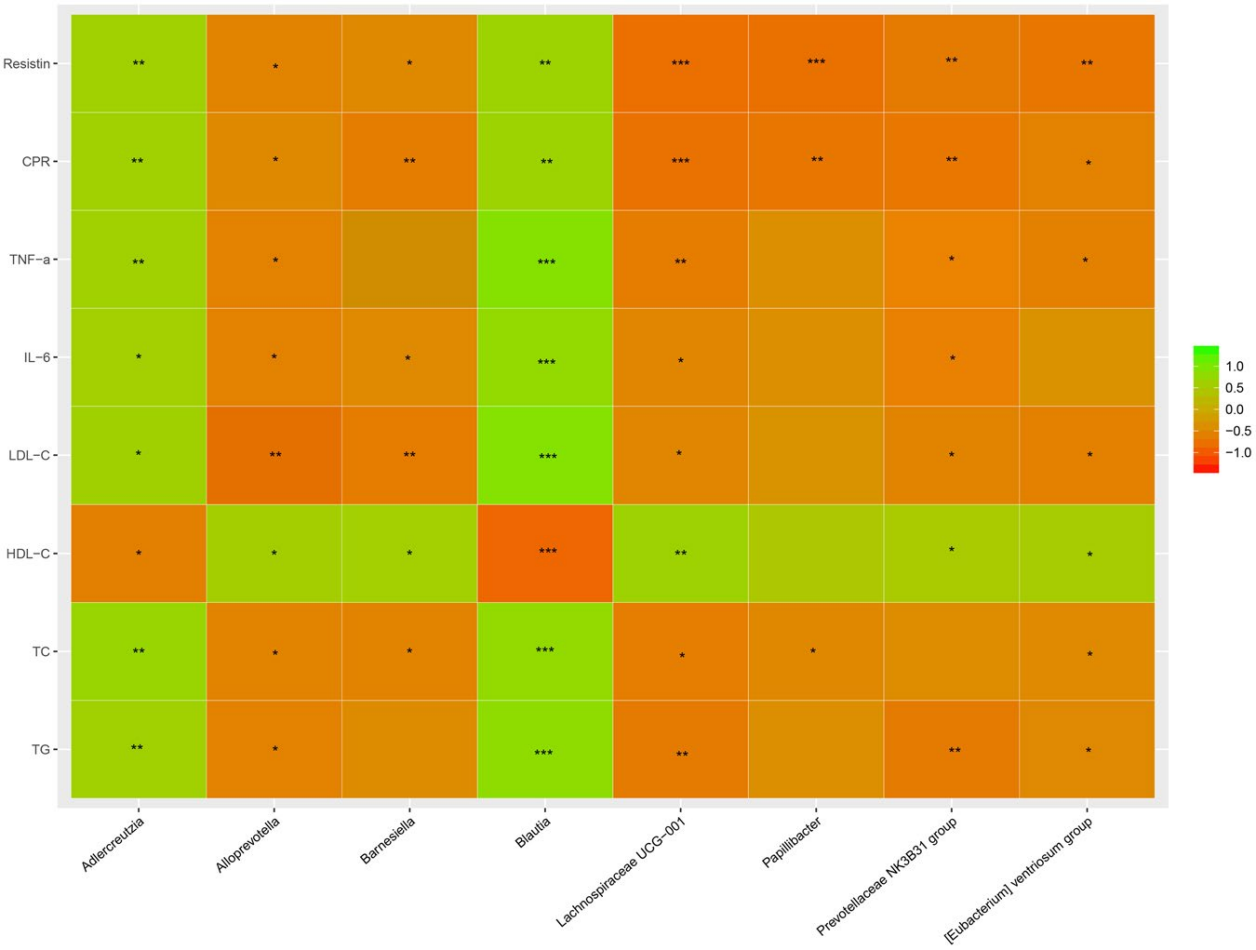
Correlation analysis between T2DM-related biochemical factors and gut microflora. Relative heat-map analysis between intestinal biomarkers and significantly affected phylotypes. The colors ranged from orange (negative correlation) to green (positive correlation). Significant correlations were noted by ^*^P < 0.05 and ^**^P < 0.01.

Multiple samples Canonical Correspondence analysis (CCA) and correlation heat-map analysis were applied to investigate the correlations between intestinal microbiota (markedly changed eight bacteria by XXT) and T2DM related indexes (TC, TG, LDL-C, HDL-C, IL-6, TNF-α, CRP and resistin) (Figs 7 and 8). In the resulting ordination plot, most of the variation (55.6%) was plotted on the first axis to separate the samples, while the second axis explained only 15.14% of the variability. Based on CCA and heat-map, we found that *Adlercreutzia* and *Blautia* were positively correlated with metabolic indexes (except HDL-C), While other bacteria (*Alloprevotella*, *Barnesiella*, [*Eubacterium*] *Ventriosum group*, *Lachnospiraceae UCG-001*, *Papillibacter* and *Prevotellaceae NK3B31 group*) were negatively associated with the expression of T2DM-related biomarkers. Among these gut microbiota, *Blautia* showed a more significant correlation with eight investigated biochemical factors.

### Short chain fatty acid (SCFA) contents in the cecum

With the definitive chromatographic conditions, four SCFAs were well-separated (Figure S2). The contents of four SCFAs in three groups were showed in Table 3. The data suggested that the contents of SCFAs in T2DM rats were decreased remarkably compared to the normal group and obviously increased in XXT-treated rats, which illustrated that XXT might improve diabetes by increasing SCFAs production.

**Table 3:** Contents of four SCFAs in the investigated colonic samples. (mean ± SD, n = 6).

Group	Contents of analytes (ug/mL)			
	acetic acid	propionic acid	isobutyric acid	butyric acid
Normal	320.04 ± 42.97	255.02 ± 11.24	69.53 ± 2.46	787.85 ± 23.31
Model	172.33 ± 24.95^##^	166.88 ± 9.17^##^	38.01 ± 3.16^##^	298.34 ± 29.73^##^
XXT	568.82 ± 45.37^**^	481.45 ± 22.16^**^	58.43 ± 6.90^**^	1144.56 ± 49.81^**^

^##^P < 0.01, ^#^P < 0.05 vs^.^ Control; ^**^P < 0.01, ^*^P < 0.05 vs. Model.

## Discussion

The intestinal microecology played crucial roles in maintaining health and fighting disease^38^. A HFD intake has been shown to trigger gut dysbiosis, increase intestinal permeability and alter gut microbiota composition. Gut microbiota was a rich source of molecules like lipopolysaccharide (LPS) and peptidoglycan, which could cause inflammation in peripheral tissues of the body when entering into the blood from the gut lumen and subsequently lead to insulin resistance and T2DM. The cross-talk among adipose tissue, insulin resistance, systemic inflammation and intestinal microbiota was of crucial importance for illustrating the underlying mechanisms of obesity-related diseases, such as insulin resistance, hyperlipidemia, chronic inflammation, diabetes, nonalcoholic fatty liver disease and atherosclerosis.

Based on the above analysis, we extrapolated whether gut microbiota was the pharmacological target to exert the hypoglycemic effect after oral administration of XXT to T2DM rats. As expected, our results showed that XXT could effectively improve the symptoms of hyperglycaemia, insulin resistance, inflammation and lipid metabolism disorders resulted from T2DM. Chao estimate indicated that the gut microbial diversity obviously reduced in T2DM rats, which was coincided with the previous researches that host disease could decrease gut microbial diversity^39^. Furthermore, XXT could notably change the composition of the intestinal microbiota in T2DM rats and particularly influence some bacteria correlated with SCFAs-production and anti-inflammation. The data suggested that XXT mainly reversed the decrease of the proportion of *Adlercreutzia* and *Blautia* and the increase of the relative abundance of *Alloprevotella*, *Barnesiella*, [*Eubacterium*] *Ventriosum group*, *Lachnospiraceae UCG-001*, *Papillibacter* and *Prevotellaceae NK3B31 group* in T2DM rats. Correlation analysis indicated that these bacteria were significantly associated with levels of TC, TG, LDL-C, HDL-C, IL-6, TNF-α, CRP and resistin.

In rats, as well as in humans, *Firmicutes* and *Bacteroidetes* were two main phyla of bacteria in the gut microbiota. It was reported that the low glomerular filtration rate could increase the abundance of *Adlercreutzia* in chronic kidney disease patients^40^. The role of *Adlercreutzia* in T2DM has not yet been explored; however, its appearance in the results of our analyses suggested it might be a future target of interest. In the *Bacteroidetes* phylum, current researches identified that genera in the family *Prevotellaceae* had inconsistent effects: some were associated with an increased risk of diabetes mellitus, while others were contrary^41–43^. For all we know, there have been no relevant researches to reveal the association between *Prevotellaceae NK3B31 group* genera and type 2 diabetes mellitus. However, it was enriched in type 2 diabetes rats and modified after the oral administration of XXT, which indicated that its relative abundance might be affected by the pathological condition of T2DM. So it might be regarded as one of the bacterial indexes in estimating the pathological status of T2DM and one of the target bacteria during the progress that XXT alleviated T2DM. *Alloprevotella*, another genus in the family *Prevotellaceae*, of Gram-negative, obligate anaerobic bacilli, had the ability to ferment carbohydrates and produce SCFAs (acetate and butyrate)^44^. A number of researches have illustrated that its abundance was negatively correlated with obesity, diabetes, cardiovascular disease and metabolic syndrome^45–47^. As a member of the family *Porphyromonadaceae* within the phylum *Bacteroidetes*, the major end products of *Barnesiella* were butyric and iso-butyric acids; while smaller amounts of succinic, propionic and acetic acids could also be produced^48^. Studies recently demonstrated that the genus *Barnesiella* was the most abundant novel classification in healthy human gut microbiome^49^, and that *Barnesiella* might have anti-inflammatory protection in mice^50^. Besides carbohydrate fermentation, the degradation of plant aromatic compounds might also be a source of SCFAs. *Flavonifractor*, which was firstly isolated from human intestine, could convert quercetin or other flavonoids into acetic and butyric acids^51^. However, flavonoids existed in nature usually as glycosides, and *Flavonifractor* was previously identified to be as accharolytic^52^. It has been reported that *Pseudoflavonifractor* and *Barnesiella*, the neighbors of *Flavonifractor*, could hydrolyze aesculin (having the similar molecular structure with flavonoid glycosides) into glucose and aesculetin^51,53^. The deglycosylating activity of these gut microflora might facilitate the flavonoid conversion. Additionally, our results also showed that 4 genera from the phylum *Firmicutes* associated with T2DM risk profile were interrelated with hypoglycemic effect of XXT. *Papillibacter*, usually as a general health indicator, belonged to *Clostridum leptum* subgroup^54^, which were common butyrate producers. Furthermore, it was also documented that the plant polyphenol methyl ethers could be degraded into acetate and other volatile fatty acids by *Papillibacter* via transferring the methyl group to various sulfides^55^. [*Eubacterium*] *Ventriosum group* as genus level of *Eubacteriaceae* family was known as butyrate-producing bacteria^56^. *Lachnospiraceae UCG-001* and *Blautia*, having contrary variation tendency among three groups according to our analysis, were both *Lachnospiraceae* family. Many bacteria of this family were known as potent SCFA producers and their proportion could be altered by high-calorie diets^57,58^. Until now, no study has examined the ability of *Lachnospiraceae UCG-001* to ameliorate T2DM. Our data illustrated that this genus might play a significantly positive role in the process of XXT improving T2DM. *Blautia*, belonging to the family *Lachnospiraceae* of phylum *Firmicutes*, could transform the undigested carbohydrates and proteins into acetic acid, and then produce energy for organism^59^. However, many studies illustrated that abundance of *Blautia* was increased in some diseases like irritable bowel syndrome, nonalcoholic fatty liver diseases, Crohn’s disease and diabetes^60,61^, which was consistent with the current study, and its relative abundance was markedly decreased in XXT-treated rats compared with T2DM rats. Some studies demonstrated that some butyrate-producing bacteria could decrease the abundance of *Blautia producta* due to the organism competing for the same substrates or forming inhibitory substances^62^. Thus, it was reasonable to suppose that changes of *Blautia* showed an opposite trend with other butyrate-producing bacteria. Furthermore, *Blautia*, especially *Blautia coccoides*, could activate the secretion of TNF-α, inflammatory cytokines to an even greater extent than LPS^63^, which might explain its higher levels in many diseases. However, further researches were needed to investigate the controversial of the role of this genus.

SCFAs (acetate, propionate and butyrate), the end products of gut microbial fermentation of indigestible dietary components, appeared to stimulate epithelial regeneration and inhibit the inflammation that was partly induced by LPS^64^. SCFAs could regulate carbohydrate and lipid metabolism in the liver, lower blood glucose and lipid levels^65,66^ and improve glucose homeostasis and insulin sensitivity by beneficially modulating the function of skeletal muscle, liver and adipose tissue^67^. Acetate and propionate could inhibit intracellular lipolysis, and propionate could also improve the lipid buffering capacity of adipose tissue by increasing lipoprotein lipase-mediated triglyceride extraction. This effect leaded to the decrease of lipid overflow and ectopic accumulation of fat, and then positively influenced insulin sensitivity. Moreover, acetate was the least potent inhibitor of histone deacetylase^68,69^, which has been found to effectively manage insulin resistance and T2DM by regulating insulin signal pathways and glucose utilization^68^. Butyrate could ameliorate epithelial barrier function and intestine permeability by regulating expression of tight junction protein and mucins, and modulate chronic low-grade inflammation via activating anti-inflammatory Treg cells and suppressing pathways involved in pro-inflammatory cytokine and chemokine production. In our research, the further investigation of cecal SCFAs suggested that treatment of XXT could increase the contents of SCFAs in T2DM rats. Moreover, correlation analysis between bacterial abundance and some T2DM-related metabolic indexes suggested that eight bacteria modulated by XXT showed a significant relationship with inflammation or lipid levels. It might be that their metabolites SCFAs possessed the capacity of strengthening anti-inflammatory pathways and regulating lipid homeostasis.

Our results indicated that XXT could improve the hyperglycemia and the metabolic disturbance of lipids and inflammation in T2DM rats. Moreover, XXT could shape the microbiome and increase the contents of cecal SCFAs, which had implications in the pathogenesis of T2DM and inflammation. Then, we supposed that the effects of XXT on T2DM rats were closely related to SCFAs-producing and anti-inflammatory bacteria, whose metabolites could enhance epithelial barrier function, improve gut permeability, inhibit the inflammation and then ameliorate insulin resistance and attenuate T2DM. Based on all the above, it was suggested that the modification of gut microbiota might be one of the proposed mechanisms to improve diabetes by XXT. Our studies provided novel insights into the roles that XXT exerted systemically hypoglycemic activities from a gut microbiota perspective, which might be helpful to further clarify its anti-diabetic mechanism *in vivo* and effectively apply to the clinical practice in treating T2DM.

## Materials and Methods

### Preparation of Xiexin Tang extracts

Rhei rhizome (RR), Scutellariae radix (SR) and Coptidis rhizome (CR) were obtained from the Nanjing Guo-yao Pharm Co. Ltd (Nanjing City, China). Distilled water was used for the extraction and preparation of samples. RR, SR and CR were mixed in a ratio of 2:1:1 and soaked with distilled water (1:15, w/v) for 8 h. The mixture was decocted for 2 h and filtered through gauze while hot gruffs were decocted twice with water (1:10, w/v) for 1.5 h. The three filtrates were concentrated to 1.0 g/mL at 60 °C.

### Induction of type 2 diabetes rats

Pathogen-free male Sprague-Dawley (SD) rats (body weight 200 ± 20) g were supplied by Zhejiang Province Experimental Animal Center (NO1610200008, permitted by SCXK 2014-0001 [Zhejiang]) and housed in a specific pathogen-free (SPF) animal laboratory. The animals were maintained in cages at 24–26 °C with 55–65% relative humidity, under a 12 h dark-light cycle, with water and respective diet available ad libitum. All the protocols used in this study were approved by the Research Ethical Committee of Nanjing University of Chinese Medicine (Nanjing, China), and the experimental procedures were carried out following the Guide for the Care and Use of Laboratory Animals.

All rats were fed with common pellet diets (corn 73.5%, wheat bran 20%, fish meal 5%, farina 1% and salt 0.5%) and allowed to acclimatize to the laboratory environment for one week before study. Animals were randomly separated into two groups: one group (n = 6) was fed on normal pellet diet and the other group (n = 20) was fed with high-fat diet (67.5% pellet diet, 10% lard, 20% sucrose and 2.5% yolk powder). After 4 weeks, rats, fed with high-fat diet, were fasted overnight (12 h) and received a single intraperitoneal injection of STZ (Sigma-Aldrich Ltd, dissolved in 0.1 M citric acid-sodium citrate buffer (pH = 4.2–4.5)) at the dose of 35 mg/kg (bw). Seventy-two hours later, rats conformed to the T2DM model as their FBG levels exceeded 11.1 mmol/L^70^.

### Experimental design

Experimental rats (control = 6 and T2DM = 12) were divided into three groups: Group 1 (N), normal rats; Group 2 (M), T2DM rats and Group 3 (XXT), XXT-treated diabetic rats (10 g/kg bw). After six weeks of treatment, all animals were in ambrosia overnight. Then, after anesthetized with chloral hydrate, blood samples of rats were collected by abdominal aorta puncture, and serum was separated by centrifugation at 4000 rpm for 10 min at 4 °C. Cecum samples were taken immediately after anatomy and collected in frozen pipes. All samples were stored at −80 °C until the assays performed.

### Biochemical analysis

Body weight was measured every four days. FBG levels of rats were determined by a glucose-meter (ONETOUCH, Ultra, Lifescan, USA). LDL-C, HDL-C, TC and TG concentrations in serum were detected using commercially available kits from Nanjing Jiancheng Bioengineering Institute (China). TNF-α, IL-6, CRP, Resistin and FINS levels were evaluated by commercial rat enzyme-linked immune sorbent assay (ELISA) kits (R & D Systems China Co. Ltd., Shanghai, China). Insulin resistance containing insulin resistance index (HOMA-IR) and insulin sensitivity index (HOMA-ISI) were calculated using the following formulas: HOMA-ISI = −Ln (FBG × FINS), HOMA-IR = FINS × FBG/22.5.

### DNA extraction and polymerase chain reaction amplification

Bacterial DNA was extracted from cecum samples using the E.Z.N.A.® Soil DNA Kit (Omega Bio-tek, Norcross, GA, U.S.). Agarose gel electrophoresis was used to assess the quality and quantity of DNA. The V3–V4 variable regions of the 16S ribosomal RNA gene were amplified by PCR using the primers 341 F (CCTAYGGGRBGCASCAG) and 806 R (GGACTACNNGGGTWTCTAAT). The amplification reactions were conducted under the following conditions: 95 °C for 2 min, followed by 25 cycles of denaturation at 95 °C for 30 s, annealing at 55 °C for 30 s, and extension at 72 °C for 30 s; and a final extension at 72 °C for 10 min. A 20 μL PCR reaction mixture contained 0.8 μL of each primer (5 μM), 0.4 μL of FastPfu Polymerase, 4 μL of 5 × FastPfu Buffer, 2 μL of 2.5 mM dNTPs, 10ng of template DNA, and deionized water. The PCR products were excised and purified using the AxyPrep DNA Gel Extraction Kit (Axygen Biosciences, Union City, CA, U.S.) following the manufacturer’s instructions and quantified using QuantiFluor™ -ST (Promega, U.S.).

### Sequencing and data analysis

Qubit®3.0 (Life Invitrogen) was used to quantify the purified PCR products and every twenty-four amplicons whose barcodes were different were mixed equally. Following Illumina’s genomic DNA library preparation procedure, Illumina Pair-End libraries were labeled with different multiplex indexing barcodes for sequencing (2 × 250) on an Illumina MiSeq platform (Shanghai BIOZERON Co., Ltd) in accordance with standard protocols. The raw reads were deposited into the National Center for Biotechnology Information Sequence Read Archive database.

Demultiplexing and quality filtering were performed by the Quantitative Insights into Microbial Ecology (QIIME) version 1.17. OTUs were clustered using UPARSE at 97% similarity (version 7.1 http://drive5.com/uparse/) and chimeric sequences were identified and removed using UCHIME. The phylogenetic affiliation of each 16S rRNA gene sequence was defined by Ribosomal Database Project (RDP) Classifier (http://rdp.cme.msu.edu/) against the silva (SSU128) 16S rRNA database with a confidence threshold of 70%^71^.

Statistical analysis of the animal experiment was by SPSS 11.5 (SPSS, Inc., Chicago, IL). Obvious differences were accepted at p values of <0.05. The rarefaction analysis based on Mothur v.1.21 was conducted to reveal the diversity by Shannon diversity indices^72^. PCoA was applied to examine dissimilarities in community composition and microbiota abundances were constructed based on the unweighted UniFrac distance metric. Furthermore, LEfSe was performed by non-parametric factorial Kruskal-Wallis (KW) sum-rank test and then we performed the linear discriminant analysis to assess effect size of each differentially abundant taxon or OTU. CCA and Heat-map analysis were conducted to investigate correlations between gut microbiota and diabetes related metabolize indexes.

### Short chain fatty acid analysis

0.1 g of cecal contents were suspended in 0.4 mL water and 0.1 mL 50% sulphuric acid, then mixtured and centrifuged at 13,200 rpm for 15 min at 4 °C. 0.4 mL of supernatant was extracted with 0.5 mL ethyl ether. The 2-ethylbutyric acid solution as internal standard was spiked into the supernatant, which was injected for analysis. The contents of SCFAs were determined using a Perkin Elmer Clarus^®^ 80 gas chromatograph (Perkin Elmer, Inc.) equipped with a flame ionization detector. A HP-5MS column (30 m × 0.250 mm × 0.25 um; Agilent Technologies Inc., CA, USA) was used for separating the SCFAs. An injection volume of 5 μL of sample was automatically injected into inlet, which was kept at 280 °C with 20:1 split ratio. The carrier gas was nitrogen at a flow rate of 2.0 mL/min. The FID and injector temperatures were 240 °C and 220 °C, respectively. The flow rates of nitrogen, hydrogen and air as auxiliary gases were 25, 40 and 400 mL/min, respectively. The column temperature program: held at 90 °C for 2 min and then heated at 25 °C/min to 250 °C (held for 5 min).

## Electronic supplementary material

Supplementary information
