# Supplementary material for: Xiexin Tang improves the symptom of type 2 diabetic rats by modulation of the gut microbiota
Source: Sci Rep. 2018 Feb 27;8:3685. doi: 10.1038/s41598-018-22094-2 (PMC5829262; doi:10.1038/s41598-018-22094-2)

Xiexin Tang improves the symptom of type 2 diabetic rats by modulation of the gut microbiota

Xiaoyan Wei, Jinhua Tao, Suwei Xiao, Shu Jiang*, Erxin Shang, Zhenhua Zhu, Dawei Qian, Jinao Duan**

Jiangsu Collaborative Innovation Center of Chinese Medicinal Resources Industrialization, Nanjing University of Chinese Medicine, 138 Xianlin Road, Nanjing 210023, PR China

*Corresponding author.

Prof. Shu Jiang

Jiangsu Collaborative Innovation Center of Chinese Medicinal Resources Industrialization, Nanjing University of Chinese Medicine, 138 Xianlin Road, Nanjing 210023, PR China

Tel. / fax:+86 25 85811516

*Correspondence to these authors.

Prof. Jin-ao Duan

Jiangsu Collaborative Innovation Center of Chinese Medicinal Resources Industrialization, Nanjing University of Chinese Medicine, 138 Xianlin Road, Nanjing 210023, PR China

Tel / fax: +86 25 85811116

E-mail: jiangshu2020@126.com (S. Jiang); dja@njutcm.edu.cn (J.A. Duan)

Figure S1. Linear discriminant analysis (LDA) score of key genera of rats' gut microbiota in each group by LEfSe analysis. The higher the score was, the more important the role was. Only taxa meeting an LDA score threshold >2 were listed. Blue = normal; Red = model; Green = Xiexin Tang, n = 5.

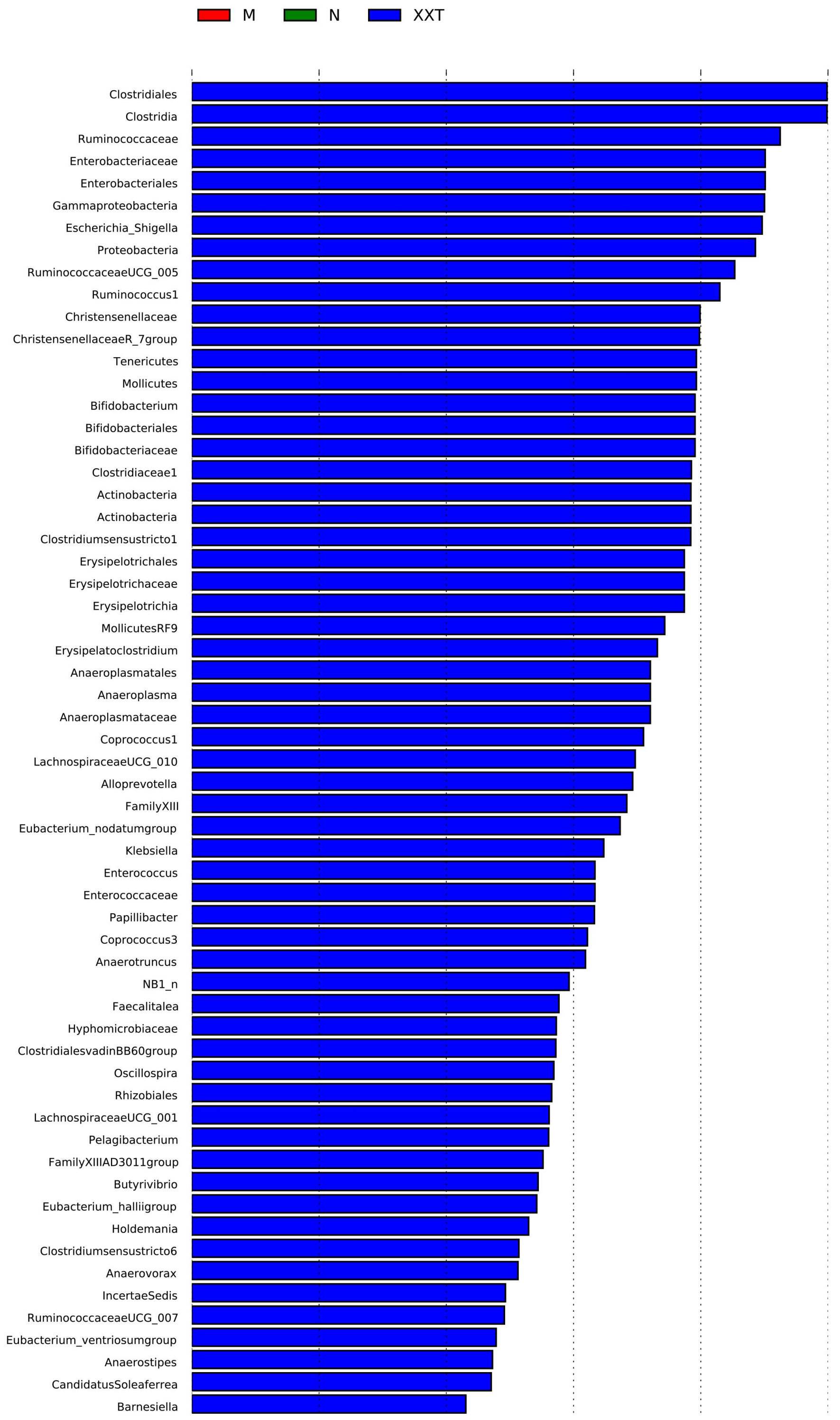

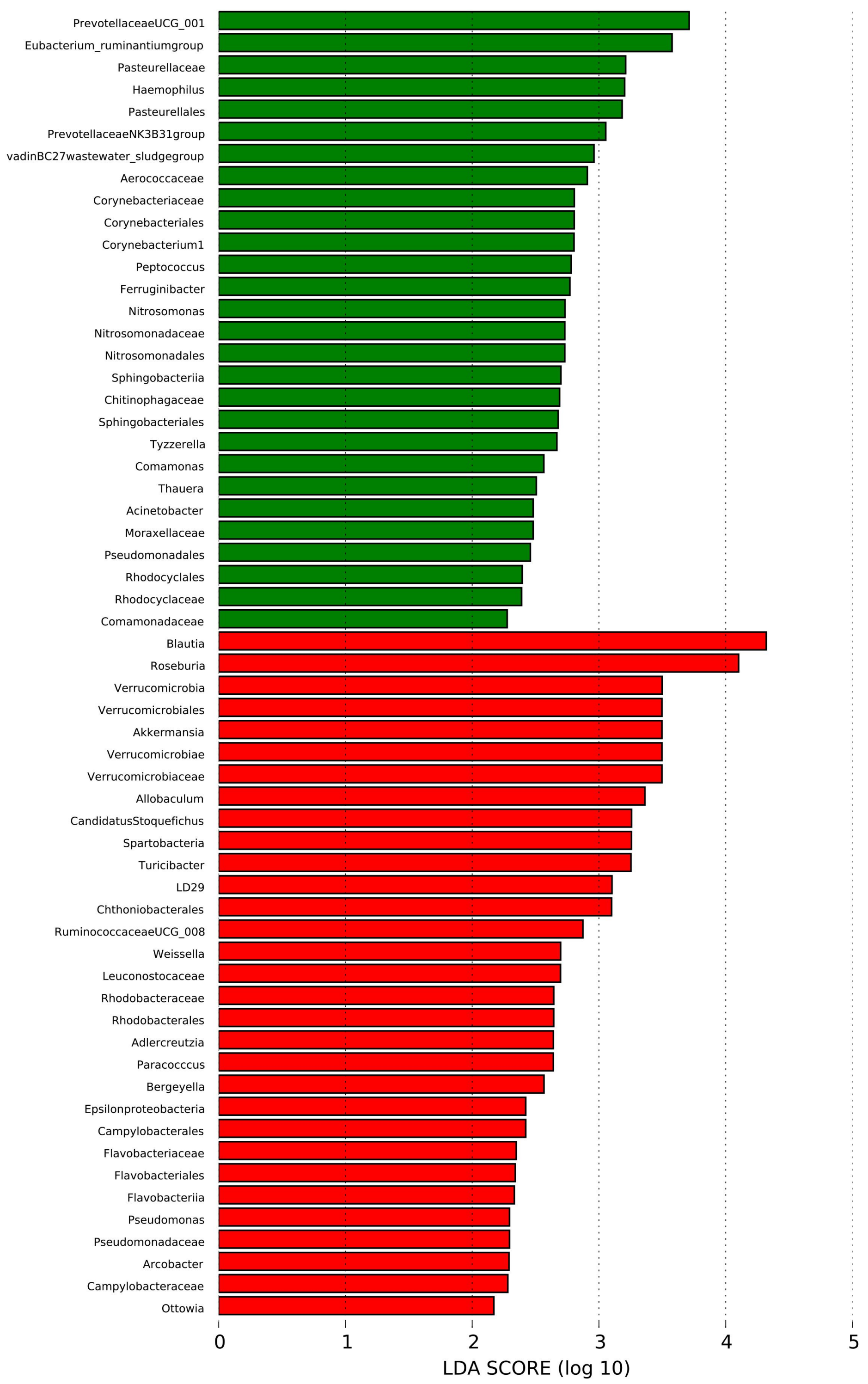
PrevotellaceaeUCG_001
Eubacterium_ruminantiumgroup
Pasteurellaceae
Haemophilus
Pasteurellales
PrevotellaceaeNK3B31group
vadinBC27wastewater_sludgegroup
Aerococcaceae
Corynebacteriaceae
Corynebacteriales
Corynebacterium1
Peptococcus
Ferruginibacter
Nitrosomonas
Nitrosomonadaceae
Nitrosomonadales
Sphingobacteriia
Chitinophagaceae
Sphingobacteriales
Tyzzerella
Comamonas
Thauera
Acinetobacter
Moraxellaceae
Pseudomonadales
Rhodocyclales
Rhodocyclaceae
Comamonadaceae
Blautia
Roseburia
Verrucomicrobia
Verrucomicrobiales
Akkermansia
Verrucomicrobiae
Verrucomicrobiaceae
Allobaculum
CandidatusStoquefichus
Spartobacteria
Turicibacter
LD29
Chthoniobacterales
RuminococcaceaeUCG_008
Weissella
Leuconostocaceae
Rhodobacteraceae
Rhodobacterales
Adlercreutzia
Paracocccus
Bergeyella
Epsilonproteobacteria
Campylobacterales
Flavobacteriaceae
Flavobacteriales
Flavobacteriia
Pseudomonas
Pseudomonadaceae
Arcobacter
Campylobacteraceae
Ottowia
0
1
2
3
4
5
LDA SCORE (log 10)

Figure S2. Gas chromatogram of short chain fatty acids. Peak identification: 1, acetic acid; 2, propionic acid; 3, isobutyric acid; 4, butyric acid; 5, 2-ethylbutyric acid. (A) standard mixture, (B) normal group, (C) T2DM group, (D) XXT-treated group.

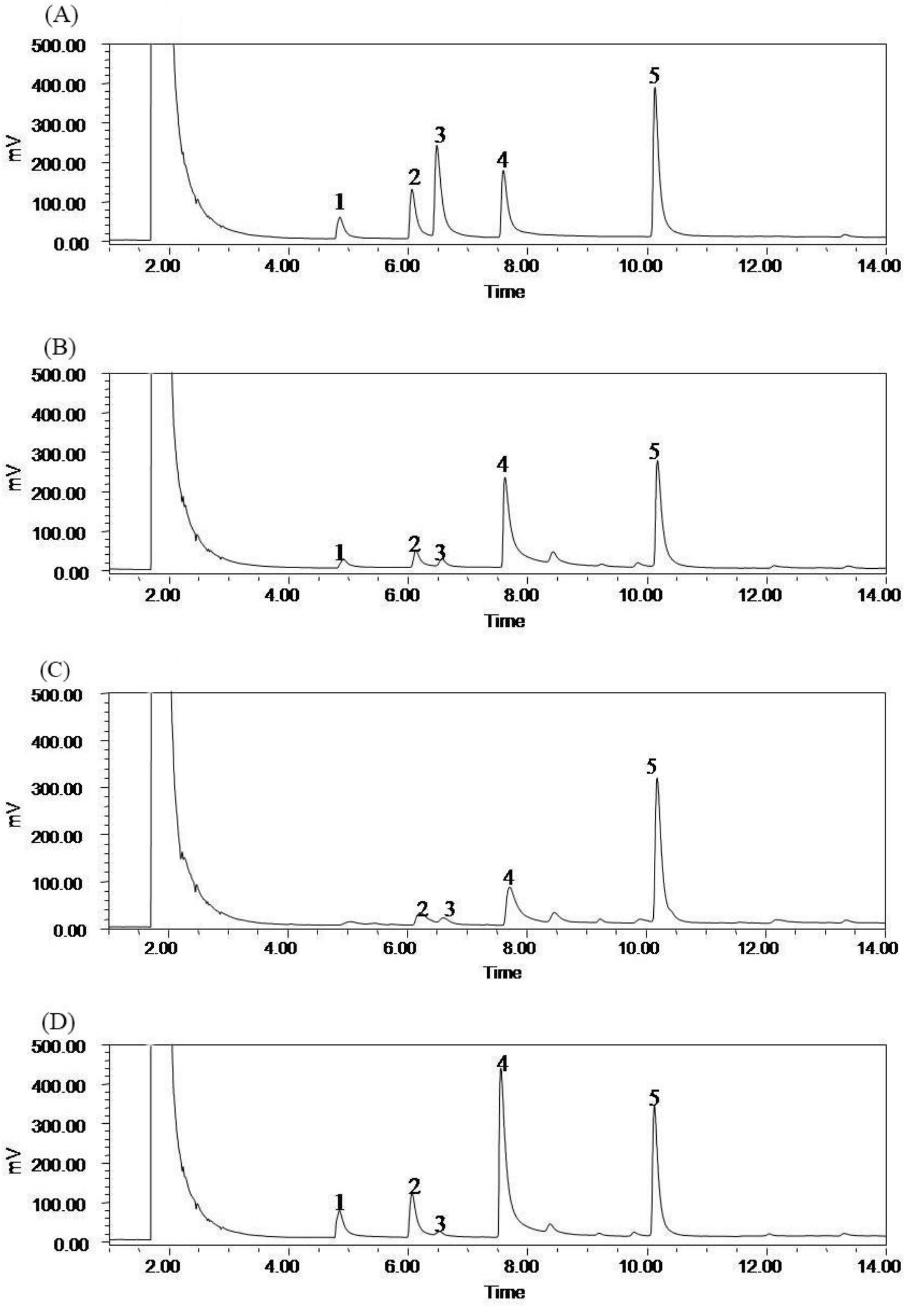

Supplement: Supplementary file 1 — Supplementary information [file 41598_2018_22094_MOESM1_ESM.pdf]
